# Long-Term Crop Rotation Improves Drought Resilience and Modulates Antioxidant Responses in Spring Wheat Leaves and Roots

**DOI:** 10.3390/plants15162535

**Published:** 2026-08-21

**Authors:** Shuli Wei, Jing Fang, Yunlong Hou, Shaofeng Su, Kun Zhao, Gongfu Shi, Rui Xie, Liyu Chen, Huimin Shi, Xiaoyu Zhao, Zhanyuan Lu, Xiaoqing Zhao

**Affiliations:** 1 Key Laboratory of Black Soil Protection and Utilization, Ministry of Agriculture and Rural Affairs, State Key Laboratory of Agricultural Ecology in the Agro-pastoral Ecotone (Cultivation), Northern Farming-Pastoral Ecotone, Farmland Ecosystem Conservation, Observation and Research Station of Inner Mongolia, Inner Mongolia Academy of Agricultural and Animal Husbandry Sciences, Hohhot 010031, China; 2 College of Agronomy, Inner Mongolia Agricultural University, Hohhot 010019, China; 3 College of Life Sciences, Inner Mongolia University, Hohhot 010030, China

**Keywords:** crop rotation, oxidative stress, redox homeostasis, osmotic adjustment, yield stability

## Abstract

Long-term crop rotation can improve soil function and crop performance, but it remains unclear whether rotation history can simultaneously alleviate drought-induced oxidative injury in spring wheat leaves and roots. To address this gap, we used a long-term field rotation experiment established in 2016 in the western foothills of the Greater Khingan Mountains. Four cropping systems were selected: spring wheat–potato rotation (R1), spring wheat–potato–rape rotation (R2), spring wheat–rape rotation (R3), and continuous spring wheat cropping (C1). In 2022, wheat occurred naturally in all rotation sequences, and all plots were planted with the same spring wheat cultivar (‘Longmai 36’) to enable comparison among different rotation histories. Drought stress was imposed from late jointing, and at anthesis, ROS-related levels, malondialdehyde (MDA), glutathione (GSH), and proline (Pro), as well as the activities of superoxide dismutase (SOD) and peroxidase (POD), were determined in the flag leaves and roots of spring wheat under normal-water (NC) and drought-stress (HC) conditions. Under drought stress, rotation significantly increased yield (R1 and R2 by 73.3% and 77.7% vs. C1, respectively, partly reflecting the low C1 baseline associated with continuous cropping) and reduced drought-induced accumulation of ROS-related levels and MDA, along with excessive antioxidant and osmotic responses. R1 better protected leaves, while R2 optimized root osmotic regulation. Two-way ANOVA revealed significant drought × rotation interactions for most leaf traits (*p* < 0.01) but not for root ROS-related levels, SOD, or POD, indicating organ-specific regulation. R1 and R2 show strong drought resilience potential, warranting multi-year, multi-site validation.

## 1. Introduction

Drought ranks among the most severe abiotic stresses limiting crop production worldwide [1]. Rising drought frequency and intensity now threaten global food security. Reported yield losses reach roughly 21% in wheat and 40% in maize, though the magnitude varies with crop species, growth stage, and stress intensity [2,3,4,5]. In the semi-arid regions of northern China, recurrent drought constrains sustainable agriculture. The western foothills of the Greater Khingan Mountains lie in a mid-temperate semi-arid continental grassland climate zone, where precipitation is sparse and unevenly distributed. In the study year (2022), annual rainfall was only 259.4 mm, with strong spring evaporation and winds that exacerbate soil water deficits. Spring wheat, the staple cereal in this region, suffers from poor synchrony between its early growth phases and rainfall patterns. Drought has become the primary bottleneck for yield and quality gains, creating an urgent need for stress-resilient cropping practices.

Drought stress triggers excessive accumulation of reactive oxygen species (ROS) in plants, leading to oxidative stress that damages membrane lipids, proteins, and nucleic acids [6]. Under normal conditions, cellular ROS—including superoxide anions, hydrogen peroxide, and hydroxyl radicals—are maintained at steady-state levels by photosynthetic and respiratory metabolism. Drought-induced stomatal closure, however, restricts CO_2_ supply and blocks photosynthetic electron transport, causing ROS production to outpace scavenging capacity [7]. The resulting oxidative burst disrupts cellular structures, suppresses photosynthesis, and inhibits growth. Plants counteract this through an elaborate antioxidant defense system comprising both enzymatic and non-enzymatic components [8,9]. The enzymatic arm centers on superoxide dismutase (SOD) and peroxidase (POD): SOD dismutates O_2_^−^ into H_2_O_2_ and O_2_, after which POD, catalase (CAT), and allied enzymes further decompose H_2_O_2_ to prevent hydroxyl radical formation. Drought-tolerant cultivars typically sustain higher SOD and POD activities to preserve redox balance [10]. The non-enzymatic arm relies on reduced glutathione (GSH), ascorbate (AsA), and related metabolites; GSH directly scavenges ROS and regenerates reducing power through the AsA-GSH cycle [11]. Proline (Pro), a compatible solute and osmotic regulator, accumulates strongly under drought, lowering osmotic potential to maintain turgor, stabilizing macromolecules, scavenging ROS, and modulating stress-responsive gene expression [12]. Malondialdehyde (MDA), a terminal product of membrane lipid peroxidation, serves as a reliable marker of cellular membrane damage; tolerant genotypes generally suppress MDA accumulation by strengthening antioxidant capacity [13,14,15]. Recent multi-omics evidence in spring wheat further showed that drought induces coordinated responses involving osmotic adjustment, oxidative stress, stomatal regulation, and rhizosphere microbial processes [16].

Crop rotation is a well-established agronomic practice that improves soil physicochemical properties, reshapes microbial communities, enhances enzyme activities and nutrient supply, and raises both yield and quality. Relative to continuous cropping, rotation interrupts pest and disease cycles, builds soil organic matter, and indirectly bolsters stress tolerance [17]. Most existing work, however, has focused on rotation effects on yield, soil fertility, or single-organ physiological traits. Studies addressing how rotation patterns regulate antioxidant defense under drought—particularly those spanning both leaves and roots—remain scarce. Gong et al. [18] showed that intercropping and rotation can delay functional leaf senescence and reduce MDA and ROS accumulation, mitigating continuous-cropping-induced lipid peroxidation. Ma et al. [19] reported that rational rotation alleviates continuous cropping obstacles and the associated oxidative damage and MDA buildup. For spring wheat specifically, no field study has directly compared how different rotation patterns modulate leaf and root antioxidant systems under drought interaction, nor quantified the relative contribution of each rotation to antioxidant defense in the two organs.

We therefore hypothesized that long-term rotation lowers ROS accumulation and lipid peroxidation in drought-stressed spring wheat, reduces reliance on antioxidant enzyme upregulation, and thereby stabilizes yield—with possible organ-specific differences between leaves and roots. This study aimed to: (1) characterize how different rotation patterns differentially regulate antioxidant enzyme activities, non-enzymatic antioxidants, and osmotic regulators in flag leaves and roots under drought; (2) quantify a drought-mitigation effect value for each physiological trait and dissect its organ specificity (leaf vs. root); and (3) explore the linkage between rotation-mediated drought relief and antioxidant defense responses, providing a theoretical basis for selecting suitable rotation patterns in the semi-arid western foothills of the Greater Khingan Mountains.

## 2. Materials and Methods

### 2.1. Site Description

The experiment was conducted at the Tenihe Soil Management and Ecological Restoration Scientific Observation Station of the Inner Mongolia Academy of Agricultural and Animal Husbandry Sciences (Tenihe Farm, 120°48′ E, 49°55′ N, 650 m a.s.l.). The site lies in a mid-temperate semi-arid continental grassland climate zone with a frost-free period of 90–105 days. In 2022, the mean annual temperature was −0.38 °C and mean annual precipitation was 259.4 mm, of which 71.13% fell between May and August. Total sunshine duration was 2828.3 h. The soil at the experimental site is classified as chernozem. Field capacity was determined using the cutting-ring method with undisturbed soil samples collected from the 0–20 cm soil layer, and the measured field capacity of the experimental soil was approximately 30–35%. Complete plot-level meteorological records for individual growing seasons during 2016–2021 were unavailable. These years were used primarily to establish the long-term rotation histories under rainfed conditions, whereas the controlled drought experiment and physiological measurements analyzed in the present study were conducted in 2022.

### 2.2. Plant Material

The spring wheat cultivar ‘Longmai 36’ was used, provided by the Tenihe Soil Management and Ecological Restoration Scientific Observation Station. This cultivar was approved by the Heilongjiang Provincial Crop Variety Approval Committee in 2013 (approval no. 2013001) and bred by the Crop Resources Research Institute, Heilongjiang Academy of Agricultural Sciences. It is characterized by high yield, good grain quality, broad adaptation, and tolerance to both drought and cold, making it suitable for cultivation in the western foothills of the Greater Khingan Mountains.

### 2.3. Experimental Design

The long-term rotation experiment was initiated in 2016. Four rotation patterns were established using spring wheat, rape, and potato as the main crops: wheat–potato (R1), wheat–potato–rape (R2), wheat–rape (R3), and continuous wheat (C1). From 2016 to 2021, all crops were grown under rainfed conditions following their rotation cycles. The annual crop sequences of the four cropping systems from 2016 to 2022 are summarized in Supplementary Table S1. Crop-specific field management practices during the rotation period, including cultivar, row spacing, seeding rate or plant spacing, and fertilizer application, are presented in Supplementary Table S2. In 2022 (the seventh year), all four cropping systems reached the wheat phase of their respective rotation cycles; therefore, all plots were sown to ‘Longmai 36’ spring wheat without altering the predefined crop sequences. Because R1 and R3 followed 2-year cycles whereas R2 followed a 3-year cycle, the accumulated frequency of wheat differed among systems. By 2022, R1 and R3 had each included four wheat seasons, R2 had included three wheat seasons, and C1 had been continuously cropped with wheat for seven seasons. Thus, the present design reflects the integrated legacy of rotation composition and crop frequency; these two components cannot be completely disentangled. Unlike the preceding rotation-establishment years (2016–2021), which were managed under rainfed conditions, controlled water treatments were introduced in 2022 for the drought-response experiment. Based on the measured field capacity of the experimental soil and established concepts of plant water stress [20], two water regimes were imposed during the 2022 wheat growing season: a normal-water control (NC), with soil water content maintained at 25–28%, and a drought-stress treatment (HC), with soil water content maintained at 9–12%. A rain shelter was used to exclude precipitation, with supplemental irrigation controlling moisture levels. The experiment followed a randomized complete block design with 8 treatments, 3 replicates each, giving 24 plots of 9 m^2^ with 1 m guard rows, totaling about 240 m^2^. Only basal fertilizer was applied (no top-dressing): urea at 60 kg ha^−1^, diammonium phosphate at 60 kg ha^−1^, and potassium sulfate at 30 kg ha^−1^. All other management followed local field practice.

At the late jointing stage (4 July 2022), rain shelters were installed over both NC and HC plots to exclude natural rainfall, marking the initiation of the controlled water treatments. Prior to this date, all plots had been maintained under rainfed conditions without supplemental irrigation. To maintain ventilation, the plastic sheeting on both sides of each shelter was kept 1.5 m above ground. From 4 July onward, soil water content in the 0–20 cm layer was measured daily in all plots. The HC plots received no irrigation throughout the drought-treatment period. In the NC plots, supplemental irrigation was applied only when the measured soil water content fell below the lower limit of the predefined normal-water range (25–28%), with each irrigation event applying 900 m^3^ ha^−1^ of water. Accordingly, C1 and R3 were irrigated on 8 and 24 July, whereas R1 and R2 were irrigated on 21 and 24 July. Daily soil-moisture measurements were also used to monitor the decline in soil water content in the HC plots. By 27 July, all HC plots had reached the predefined drought-stress range of 9–12%. At physiological sampling, the mean soil water contents of HCC1, HCR1, HCR2, and HCR3 were 9.54%, 10.41%, 10.70%, and 10.12%, respectively, and all remained within the predefined drought range. All eight treatments were sampled simultaneously at anthesis beginning on 28 July 2022.

### 2.4. Measurements

Agronomic traits and root phenotype: In each plot, representative plants of uniform growth were selected. Plant height was measured with a ruler, and whole-plant fresh weight was recorded with an electronic balance (BSA822, Sartorius AG, Göttingen, Germany). Plants were then killed at 105 °C for 20 min and dried to constant weight at 80 °C for dry weight determination. Roots within the 0–20 cm soil layer were excavated, washed, and scanned with a transmission scanner (Epson V700, Seiko Epson Corporation, Suwa, Japan); images were analyzed using WinRHIZO Pro 2009a for root length and root surface area. Yield and components: At maturity, a 1 m^2^ uniform sample segment per plot was harvested for yield component analysis—spike number per unit area, grain number per spike, and 1000-grain weight were recorded, and theoretical yield was calculated. Whole-plot harvest was also conducted to determine actual grain yield.

Flag leaf and root physiological indicators: Fifteen uniform, representative spring wheat plants were selected per plot. Flag leaves and roots were cut with sterilized stainless-steel scissors, washed, blotted dry, placed in sterilized sampling tubes, labeled, snap-frozen in liquid nitrogen, and stored at −80 °C for antioxidant enzyme assays. The flag leaves and roots from 15 plants per plot were pooled as one biological replicate, with three plots per treatment giving three replicates (n = 3). For extraction, 0.5 g of fresh material was ground in an ice bath with 5 mL of pre-cooled pH 7.8 phosphate buffer (containing 1 mmol L^−1^ EDTA, 1% PVP, and 0.5% Triton X-100), centrifuged at 4 °C and 12,000× *g* for 15 min, and the supernatant was retained for analysis. Reactive oxygen species (ROS)-associated antigen levels were determined using a plant ROS ELISA kit (Shanghai Youshuo Biotechnology Co., Ltd. (Shanghai, China), cat. no. YX-181519P, detection range 15–480 U mL^−1^, intra-assay CV < 8%) according to the manufacturer’s instructions. This kit uses a double-antibody sandwich method, with absorbance read at 450 nm and ROS-related antigen concentration (U mL^−1^) calculated from a standard curve. Reduced glutathione (GSH) content was determined using a plant glutathione ELISA kit (Shanghai Lianmai Biological Engineering Co., Ltd. (Shanghai, China), cat. no. LM-12211-ES) according to the manufacturer’s instructions. ELISA was chosen for its high standardization, good inter-batch reproducibility, and capacity for simultaneous high-throughput sample processing. It should also be noted that ROS-related levels in this study were determined using a commercial ELISA kit. This approach differs in detection principle from NBT/DAB staining and fluorescent-probe assays; therefore, the resulting values should be interpreted as ROS-related levels rather than direct measurements of instantaneous concentrations of specific ROS. This methodological limitation is considered further in the Discussion.

Malondialdehyde (MDA) content was determined by the thiobarbituric acid colorimetric method, following Heath and Packer, with modifications to correct for interfering compounds in plant tissues; absorbance was read at 450, 532, and 600 nm [21,22]. Superoxide dismutase (SOD) activity was measured by the NBT photochemical reduction method at 560 nm, with one unit defined as the enzyme amount inhibiting NBT photoreduction by 50% [23]. Peroxidase (POD) activity was assayed by the guaiacol colorimetric method, recording absorbance changes at 470 nm [24]. Proline (Pro) content was determined by the acidic ninhydrin method at 520 nm against a proline standard curve [25].

### 2.5. Drought Mitigation Effect Value

Compared with normal water conditions (NC), drought stress significantly increased or decreased each physiological and agronomic trait across the four patterns. The magnitude of this change was expressed as a percentage. The drought mitigation effect value for each rotation pattern (R1, R2, R3) was then calculated as the reduction in its change percentage relative to that of C1. Letting *PC*_1_ denote the change percentage for C1, and *PR*_1_, *PR*_2_, *PR*_3_ the corresponding values for R1, R2, R3, the drought mitigation effect value for each rotation pattern was calculated as Equation (1):

*ER*_1_ = [(*PC*_1_ − *PR*_1_)/*PC*_1_] × 100% (1)

For traits that increased under drought (e.g., ROS, MDA, antioxidant enzyme activities, and osmotic regulators), a larger effect value indicates a smaller drought-induced increment under rotation, meaning lighter oxidative or osmotic stress—not weaker antioxidant capacity. For traits that decreased under drought (e.g., yield, biomass), a larger effect value indicates smaller yield loss or growth inhibition. When NC and HC groups within a given rotation pattern showed no significant difference (*p* > 0.05), the drought effect itself was considered weak, the mitigation effect value was not applicable, and the entry was marked with “—” in the table.

### 2.6. Statistical Analysis

Statistical analyses, except for correlation analysis, were performed using SPSS Statistics (version 26.0). Data are presented as mean ± standard deviation (SD), where applicable. Before analysis of variance, the normality of model residuals was assessed using the Shapiro–Wilk test, and homogeneity of variances was evaluated using Levene’s test. Two-way analysis of variance (ANOVA) was performed to evaluate the effects of water treatment (D), rotation pattern (R), and their interaction (D × R) on each measured trait. For the 2 × 4 factorial design with three independent plot-level replicates per treatment combination, the degrees of freedom were 1 for D, 3 for R, 3 for D × R, and 16 for the residual error. The overall main effect of water treatment (NC vs. HC), averaged across rotation patterns, was assessed using the D term of the two-way ANOVA, and its significance is indicated by symbols above the brackets in the corresponding figures. For multiple comparisons among the eight drought–rotation treatment combinations (NCC1, NCR1, NCR2, NCR3, HCC1, HCR1, HCR2, and HCR3), Duncan’s multiple range test was used at a significance level of *p* < 0.05. Different lowercase letters in the figures indicate significant differences among the eight treatment combinations according to Duncan’s multiple range test (*p* < 0.05). Pearson correlation coefficients and their corresponding *p* values were calculated in R (version 4.5.2) using plot-level observations pooled across the water regimes and rotation patterns (n = 24). Correlation heatmaps were generated using the ggplot2 package (version 4.0.0) in R. Statistical significance was determined at *p* < 0.05, with *, **, and *** indicating *p* < 0.05, *p* < 0.01, and *p* < 0.001, respectively. Other figures were generated using GraphPad Prism (version 9.0).

## 3. Results

### 3.1. Agronomic Traits and Root Phenotype Under Drought Stress and Rotation Patterns

**Figure 1:**
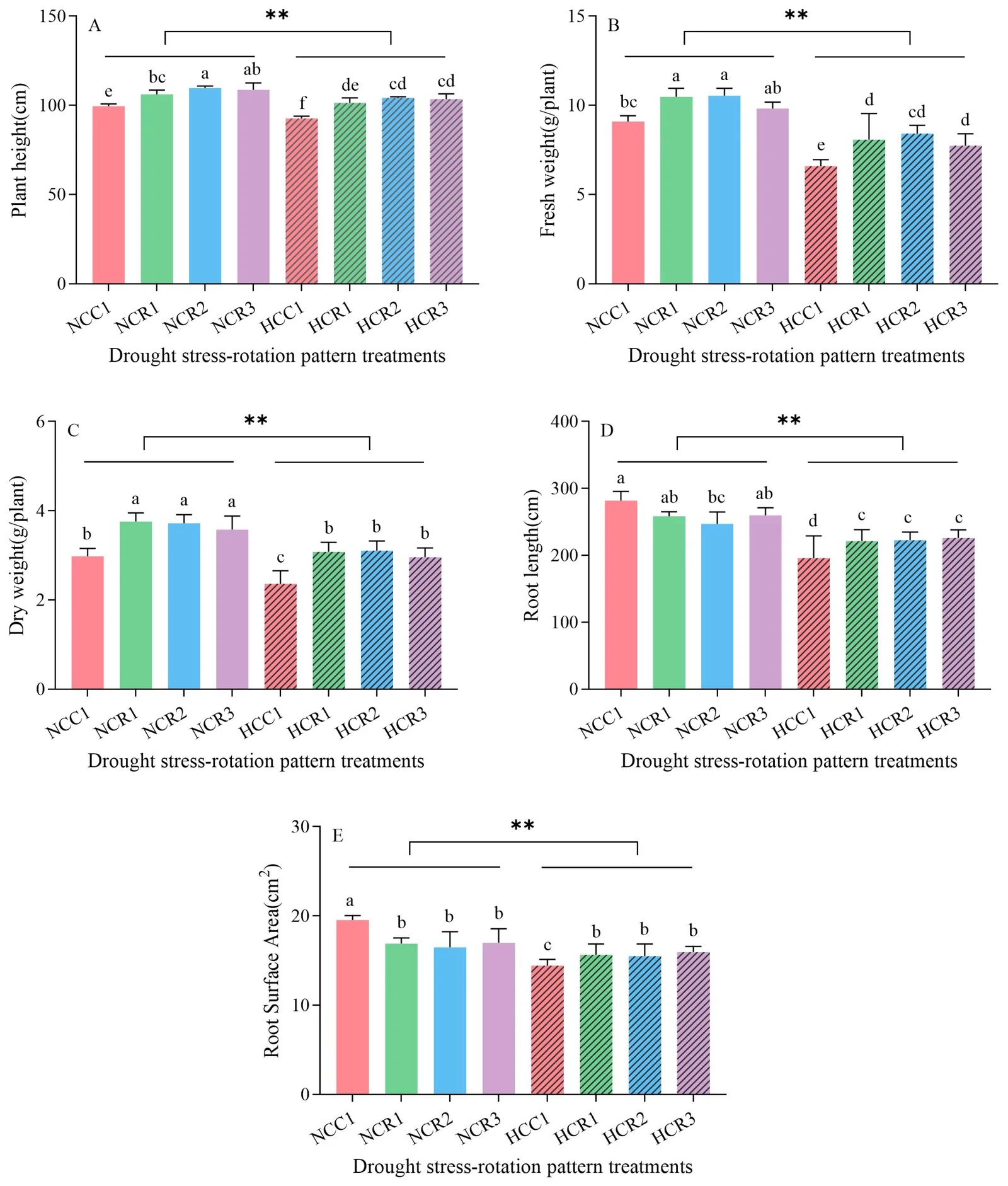
Plant and root phenotypic traits of spring wheat under drought stress and rotation patterns: (**A**) plant height (PH), (**B**) fresh weight (FW), (**C**) dry weight (DW), (**D**) root length (RL), and (**E**) root surface area (RSA). NC group: NCC1, NCR1, NCR2, NCR3; HC group: HCC1, HCR1, HCR2, HCR3. Values are presented as mean ± SD (n = 3 independent biological replicates). Different lowercase letters denote significant differences among the eight drought–rotation treatment combinations according to Duncan’s multiple range test (*p* < 0.05). Asterisks above the brackets indicate the overall main effect of water treatment (D; NC vs. HC), averaged across rotation patterns, according to two-way ANOVA; ** indicates *p* < 0.01.

Drought stress significantly reduced plant height, fresh weight, and dry weight of spring wheat (*p* < 0.01), while rotation markedly alleviated this inhibition (Figure 1). Under both NC and HC, plant height and dry weight of R1, R2, and R3 were significantly higher than continuous cropping C1 (*p* < 0.05): under drought, R1, R2, and R3 exceeded C1 by 9.40%, 12.29%, and 11.69% in height and by 30.51%, 31.61%, and 25.42% in dry weight. Compared with NC, C1 showed the greatest drought-induced declines in height and dry weight (6.83% and 20.81%, respectively), whereas rotation patterns declined less. R1 provided the strongest mitigation for plant height and R2 for fresh and dry weights, with drought mitigation effect values of 35.13%, 26.79%, and 20.66%, respectively. ANOVA showed that drought (D) and rotation (R) each had highly significant main effects on plant height, fresh weight, and dry weight (*p* < 0.01), while the D × R interaction was not significant for these traits (Table 1).

For root phenotype, drought stress significantly reduced root length and root surface area (*p* < 0.01). Under drought, root length of R1, R2, and R3 exceeded C1 by 18.97%, 19.69%, and 21.47% (*p* < 0.05). Compared with NC, C1 showed the largest root length decline (33.93%), while R2 declined least (9.72%), yielding a drought mitigation effect of 71.35%. Root surface area showed a water-dependent pattern: under NC, R1, R2, and R3 were 14.73–18.38% lower than C1, whereas under HC they were 13.76–16.04% higher (*p* < 0.05). Drought reduced root surface area significantly only in C1; rotation patterns showed no significant change. ANOVA indicated highly significant drought effects on root length and surface area (*p* < 0.01), a non-significant rotation main effect, but a highly significant D × R interaction (*p* < 0.01), showing that rotation’s regulation of root phenotype depends on water conditions (Table 1). Multiple comparisons among the eight drought–rotation treatment combinations showed that root surface area was significantly higher in C1 than in R1, R2, and R3 under NC (*p* < 0.05), whereas under HC, root surface area was significantly higher in R1, R2, and R3 than in C1 (*p* < 0.05), with no significant differences among R1, R2, and R3. This water-dependent rotation difference is consistent with the significant D × R interaction.

### 3.2. Yield and Yield Components Under Drought Stress and Rotation Patterns

**Figure 2:**
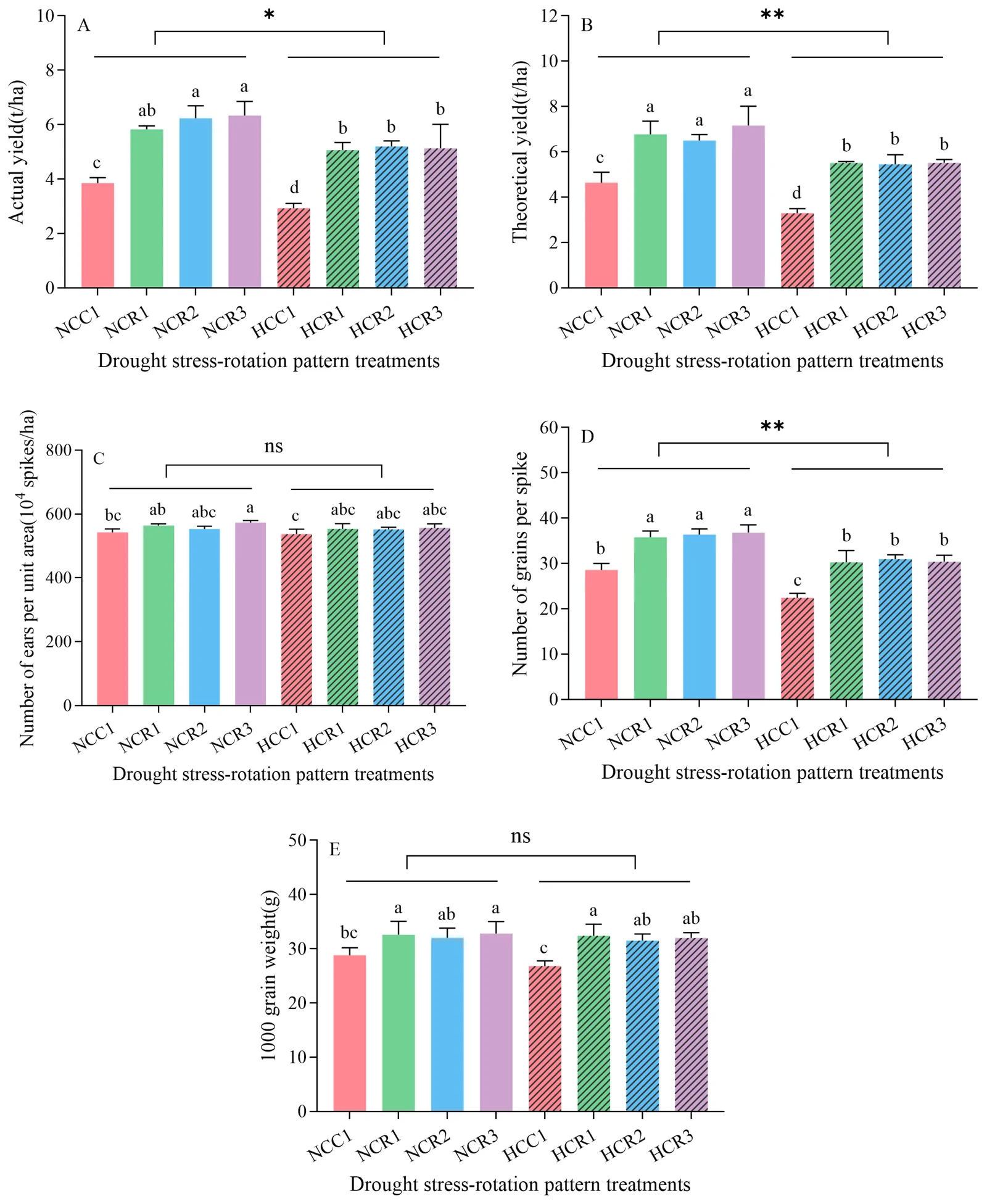
Yield and yield components of spring wheat under drought stress and rotation patterns: (**A**) actual yield (AY), (**B**) theoretical yield (TY), (**C**) spike number per unit area (SN), (**D**) grain number per spike (GN), and (**E**) 1000-grain weight (WTG). NC group: NCC1, NCR1, NCR2, NCR3; HC group: HCC1, HCR1, HCR2, HCR3. Values are presented as mean ± SD (n = 3 independent biological replicates). Different lowercase letters denote significant differences among the eight drought–rotation treatment combinations according to Duncan’s multiple range test (*p* < 0.05). Symbols above the brackets indicate the overall main effect of water treatment (D; NC vs. HC), averaged across rotation patterns, according to two-way ANOVA; ns, *, and ** indicate *p* > 0.05, *p* < 0.05, and *p* < 0.01, respectively.

Rotation significantly increased spring wheat yield and effectively mitigated drought-induced yield loss (Figure 2). Under both NC and HC, actual and theoretical yields of R1, R2, and R3 were significantly higher than C1 (*p* < 0.05), with no significant differences among the three rotation patterns. Under drought, actual yields of R1, R2, and R3 exceeded C1 by 73.26%, 77.67%, and 75.38%, respectively. This large increment partly reflects the low yield baseline of C1 under long-term continuous cropping obstacles; the difference therefore captures both the positive effect of rotation and the negative effect of continuous cropping, and should not be extrapolated directly to a general farmland rotation benefit. Compared with NC, drought reduced C1 actual yield by 24.13%, while R1 declined least (12.95%), giving a drought mitigation effect of 46.33% by Equation (1). For theoretical yield, R2 declined least (16.12%), with a mitigation effect of 44.57%. Among yield components, drought significantly reduced grain number per spike (*p* < 0.05) but did not significantly affect spike number per unit area or 1000-grain weight. Rotation patterns showed significantly higher grain number per spike and 1000-grain weight than continuous cropping (*p* < 0.05): under drought, R1, R2, and R3 exceeded C1 by 35.12–38.24% in grain number and by 17.54–20.90% in 1000-grain weight. ANOVA showed highly significant rotation main effects on actual yield, theoretical yield, spike number, grain number per spike, and 1000-grain weight (*p* < 0.01); the D × R interactions were not significant for these yield-related traits (Table 2).

### 3.3. ROS-Related Levels in Leaves and Roots Under Drought Stress and Rotation Patterns

**Figure 3:**
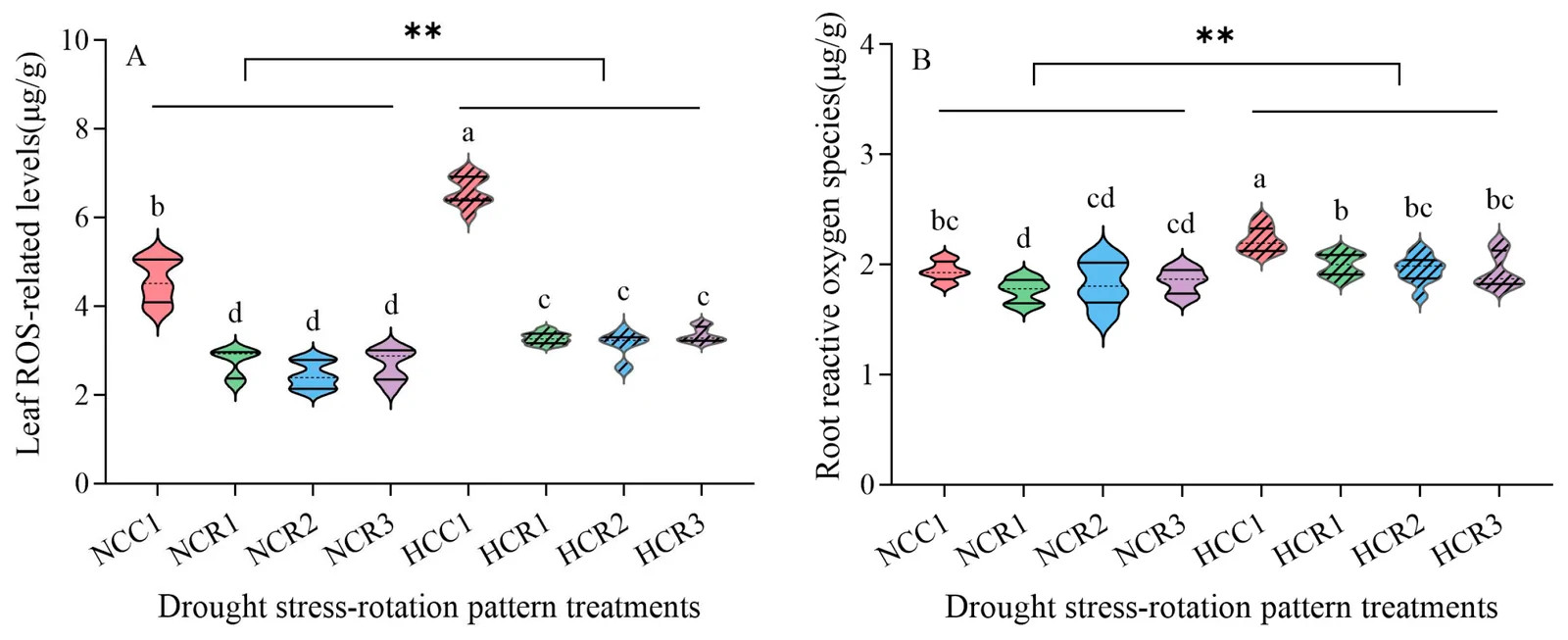
ROS-related levels in flag leaves and roots of spring wheat: (**A**) leaf ROS-related levels (L-ROS) and (**B**) root ROS-related levels (R-ROS). NC group: NCC1, NCR1, NCR2, NCR3; HC group: HCC1, HCR1, HCR2, HCR3. Each treatment consisted of three independent biological replicates (n = 3), with each biological replicate measured in technical triplicate. The width of each violin represents the kernel density distribution of the measurements. The central horizontal line indicates the median, whereas the lower and upper horizontal lines indicate the first (25th percentile) and third (75th percentile) quartiles, respectively. Different lowercase letters denote significant differences among the eight drought–rotation treatment combinations according to Duncan’s multiple range test (*p* < 0.05). Asterisks above the brackets indicate the overall main effect of water treatment (D; NC vs. HC), averaged across rotation patterns, according to two-way ANOVA; ** indicates *p* < 0.01.

**Figure 4:**
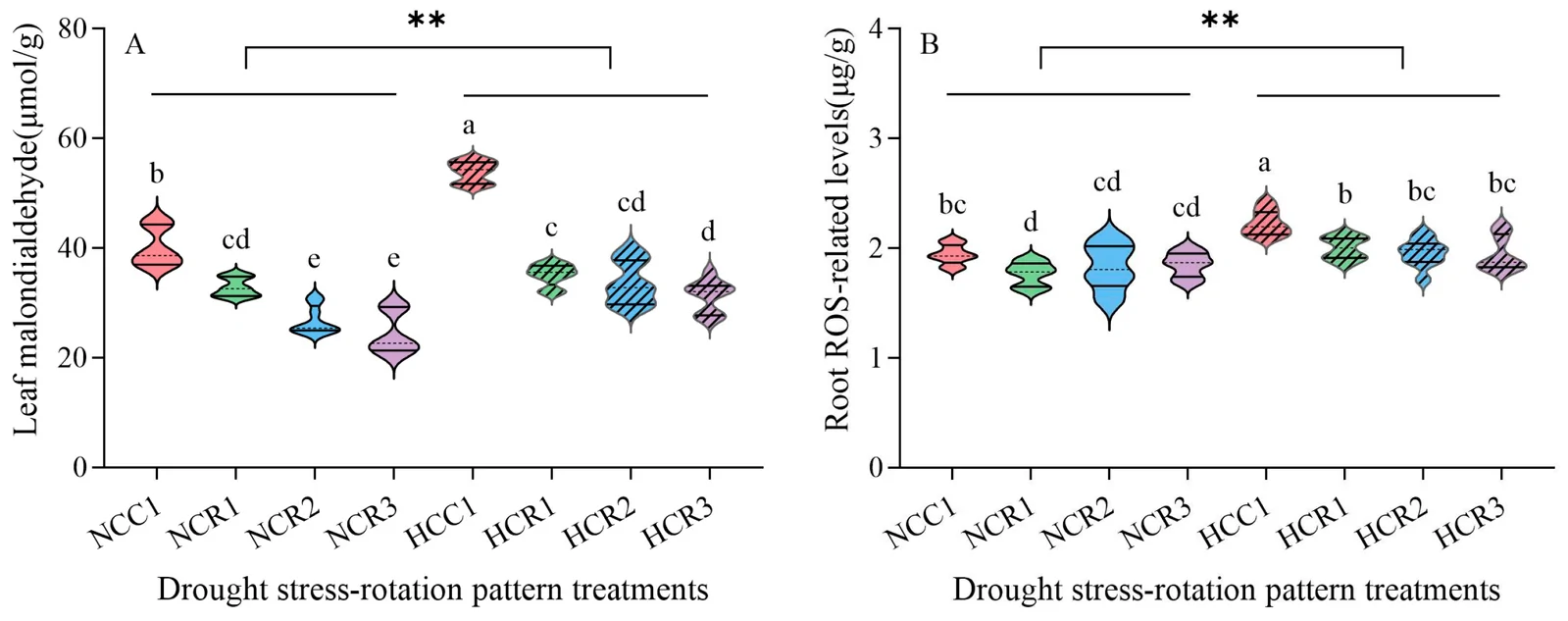
Malondialdehyde content in flag leaves and roots of spring wheat: (**A**) leaf MDA (L-MDA) and (**B**) root MDA (R-MDA). NC group: NCC1, NCR1, NCR2, NCR3; HC group: HCC1, HCR1, HCR2, HCR3. Each treatment consisted of three independent biological replicates (n = 3), with each biological replicate measured in technical triplicate. The width of each violin represents the kernel density distribution of the measurements. The central horizontal line indicates the median, whereas the lower and upper horizontal lines indicate the first (25th percentile) and third (75th percentile) quartiles, respectively. Different lowercase letters denote significant differences among the eight drought–rotation treatment combinations according to Duncan’s multiple range test (*p* < 0.05). Asterisks above the brackets indicate the overall main effect of water treatment (D; NC vs. HC), averaged across rotation patterns, according to two-way ANOVA; ** indicates *p* < 0.01.

Drought stress significantly increased ROS-related levels and MDA contents in both flag leaves and roots (*p* < 0.01), while rotation patterns were significantly lower than C1 under both water conditions (*p* < 0.05). For flag leaf ROS, R1, R2, and R3 were 40–47% lower than C1 under NC and 49–53% lower under HC. The drought-induced increment was smallest in R1 (19.55% vs. 43.67% in C1), giving a drought mitigation effect of 55.23% (Figure 3A). For root ROS-related levels, R2 and R3 showed no significant difference between NC and HC (Figure 3B). Flag leaf MDA followed a similar trend: C1 showed the largest drought-induced increment (34.43%), while R1 showed no significant difference between NC and HC, providing the best mitigation (Figure 4A). For root MDA, rotation patterns were 23–46% lower than C1; under HC, C1 increased by 38.21%, with R1 and R2 showing the most pronounced drought resistance (Figure 4B). Overall, R1 performed best in regulating leaf ROS-related levels and MDA, while R2 and R3 showed greater advantage in buffering root ROS-related levels.

### 3.4. Enzymatic Antioxidant System in Leaves and Roots Under Drought Stress and Rotation Patterns

**Figure 5:**
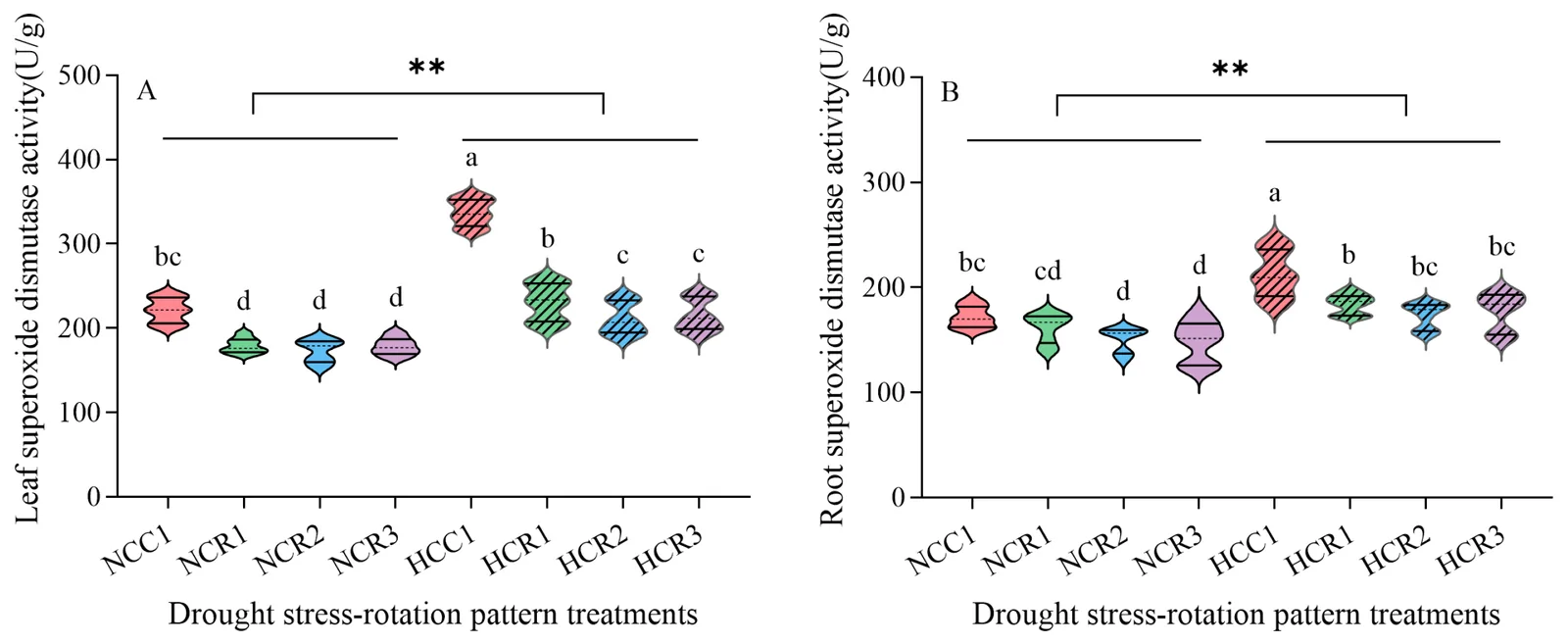
Superoxide dismutase activity in flag leaves and roots of spring wheat: (**A**) leaf SOD activity (L-SOD) and (**B**) root SOD activity (R-SOD). NC group: NCC1, NCR1, NCR2, NCR3; HC group: HCC1, HCR1, HCR2, HCR3. Each treatment consisted of three independent biological replicates (n = 3), with each biological replicate measured in technical triplicate. The width of each violin represents the kernel density distribution of the measurements. The central horizontal line indicates the median, whereas the lower and upper horizontal lines indicate the first (25th percentile) and third (75th percentile) quartiles, respectively. Different lowercase letters denote significant differences among the eight drought–rotation treatment combinations according to Duncan’s multiple range test (*p* < 0.05). Asterisks above the brackets indicate the overall main effect of water treatment (D; NC vs. HC), averaged across rotation patterns, according to two-way ANOVA; ** indicates *p* < 0.01.

**Figure 6:**
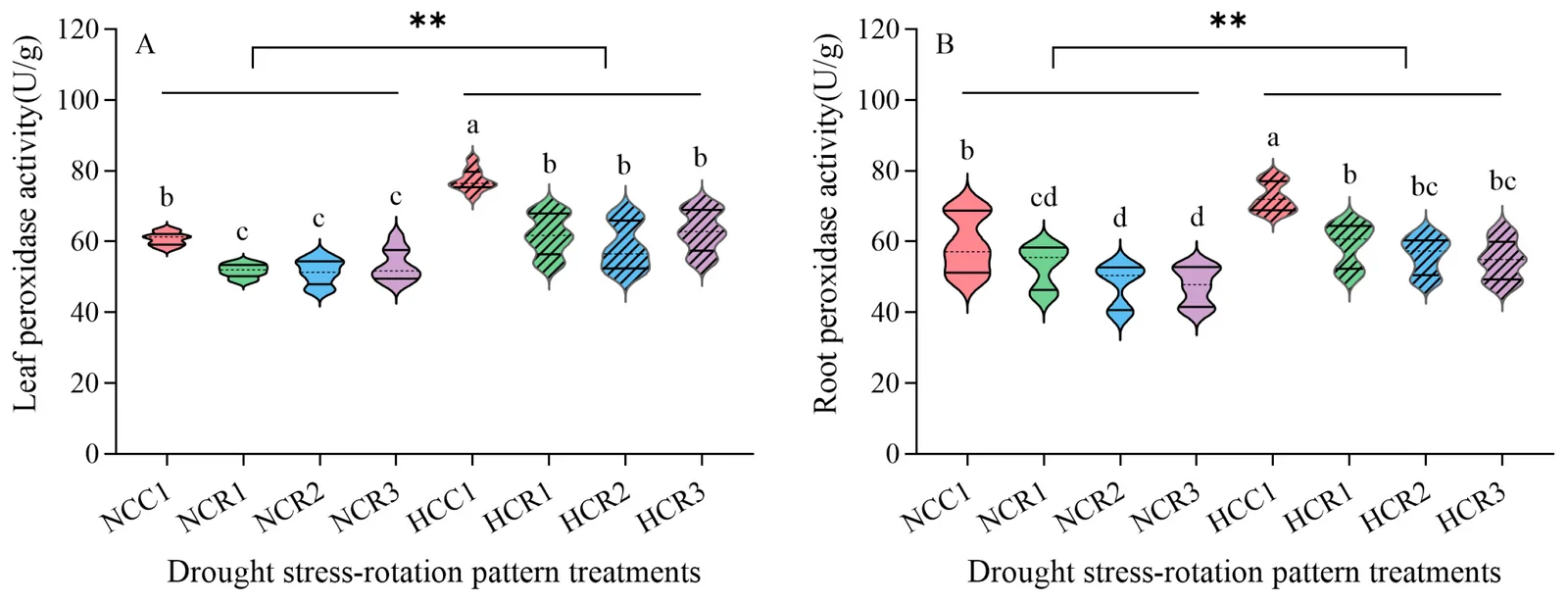
Peroxidase activity in flag leaves and roots of spring wheat. (**A**) leaf POD activity (L-POD), and (**B**) root POD activity (R-POD). NC group: NCC1, NCR1, NCR2, NCR3; HC group: HCC1, HCR1, HCR2, HCR3. Each treatment consisted of three independent biological replicates (n = 3), with each biological replicate measured in technical triplicate. The width of each violin represents the kernel density distribution of the measurements. The central horizontal line indicates the median, whereas the lower and upper horizontal lines indicate the first (25th percentile) and third (75th percentile) quartiles, respectively. Different lowercase letters denote significant differences among the eight drought–rotation treatment combinations according to Duncan’s multiple range test (*p* < 0.05). Asterisks above the brackets indicate the overall main effect of water treatment (D; NC vs. HC), averaged across rotation patterns, according to two-way ANOVA; ** indicates *p* < 0.01.

Drought stress significantly increased SOD and POD activities in both flag leaves and roots (*p* < 0.01), but both the absolute enzyme activity levels and drought-induced increments under rotation were significantly lower than in C1 (*p* < 0.05). For flag leaf SOD, R1, R2, and R3 were 19–22% lower than C1 under NC, with the gap widening to 31–38% under HC. The drought-induced increment was smallest in R3 (20.48% vs. 52.38% in C1), giving a mitigation effect of 60.90% (Figure 5A). For root SOD, R1 showed the smallest increment (13.86% vs. 23.56% in C1), with a mitigation effect of 41.17% (Figure 5B). For flag leaf POD, R1, R2, and R3 did not differ among themselves but were all significantly lower than C1; the smallest drought-induced increment was in R2 (13.89% vs. 27.17% in C1), yielding a mitigation effect of 48.88% (Figure 6A). Root POD followed a similar pattern, with R1 showing the smallest increment (11.41% vs. 22.07% in C1) and a mitigation effect of 48.30% (Figure 6B). Both enzymatic indicators showed that rotation patterns generally had smaller drought-induced increments in antioxidant enzyme activity, suggesting relatively lighter oxidative stress.

### 3.5. Osmotic Regulators in Leaves and Roots Under Drought Stress and Rotation Patterns

**Figure 7:**
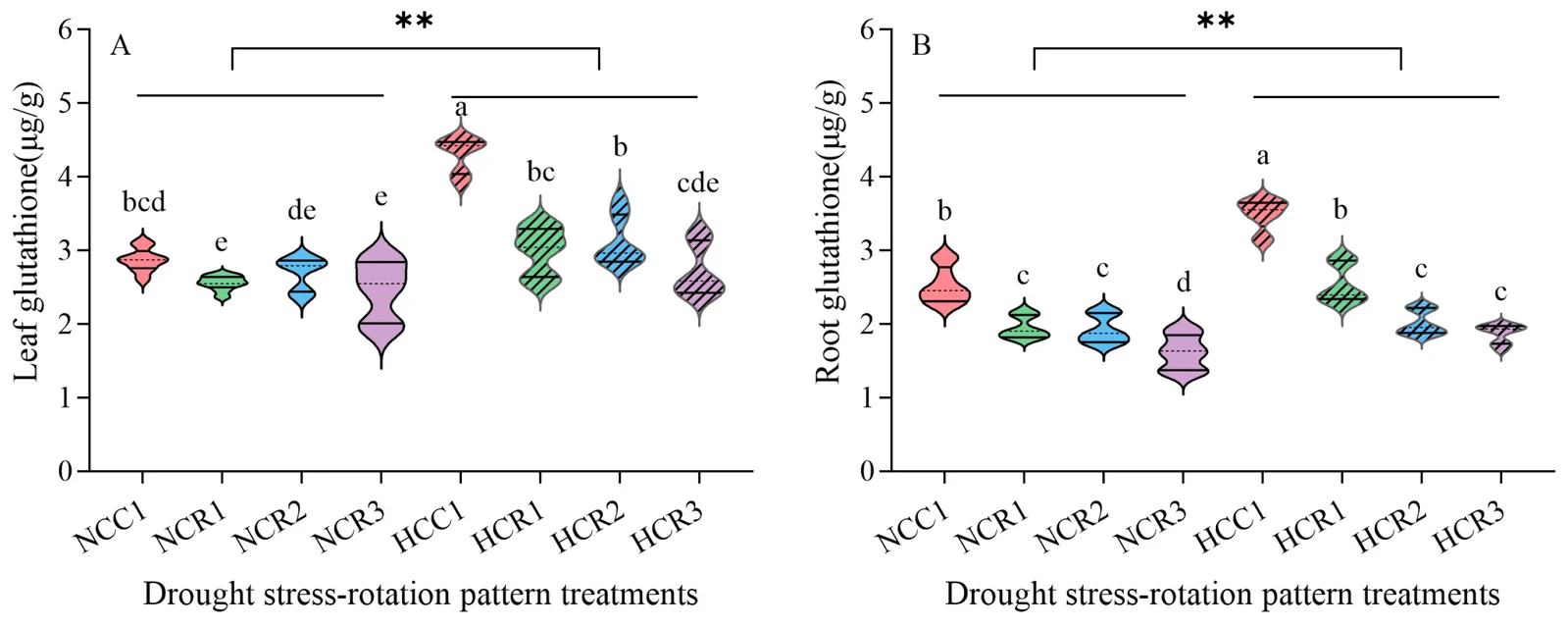
Reduced glutathione content in flag leaves and roots of spring wheat: (**A**) leaf GSH content (L-GSH) and (**B**) root GSH content (R-GSH). NC group: NCC1, NCR1, NCR2, NCR3; HC group: HCC1, HCR1, HCR2, HCR3. Each treatment consisted of three independent biological replicates (n = 3), with each biological replicate measured in technical triplicate. The width of each violin represents the kernel density distribution of the measurements. The central horizontal line indicates the median, whereas the lower and upper horizontal lines indicate the first (25th percentile) and third (75th percentile) quartiles, respectively. Different lowercase letters denote significant differences among the eight drought–rotation treatment combinations according to Duncan’s multiple range test (*p* < 0.05). Asterisks above the brackets indicate the overall main effect of water treatment (D; NC vs. HC), averaged across rotation patterns, according to two-way ANOVA; ** indicates *p* < 0.01.

**Figure 8:**
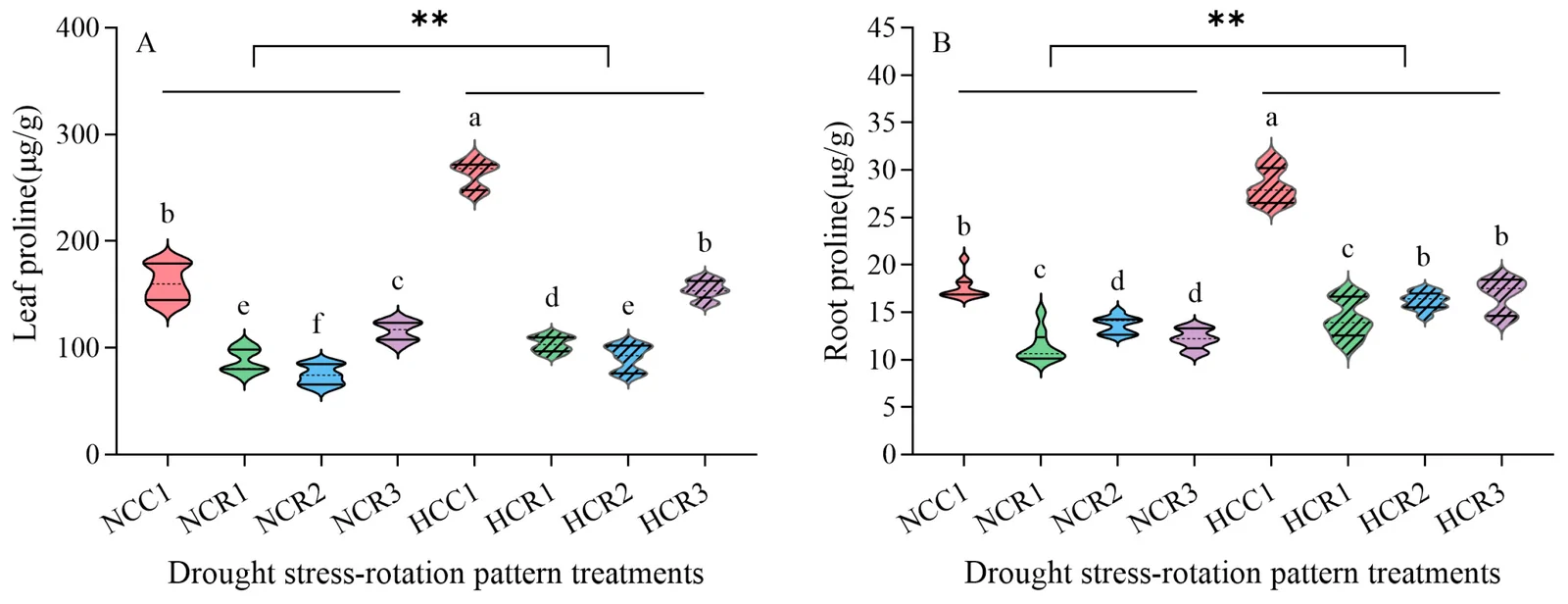
Proline content in flag leaves and roots of spring wheat: (**A**) leaf Pro content (L-Pro) and (**B**) root Pro content (R-Pro). NC group: NCC1, NCR1, NCR2, NCR3; HC group: HCC1, HCR1, HCR2, HCR3. Each treatment consisted of three independent biological replicates (n = 3), with each biological replicate measured in technical triplicate. The width of each violin represents the kernel density distribution of the measurements. The central horizontal line indicates the median, whereas the lower and upper horizontal lines indicate the first (25th percentile) and third (75th percentile) quartiles, respectively. Different lowercase letters denote significant differences among the eight drought–rotation treatment combinations according to Duncan’s multiple range test (*p* < 0.05). Asterisks above the brackets indicate the overall main effect of water treatment (D; NC vs. HC), averaged across rotation patterns, according to two-way ANOVA; ** indicates *p* < 0.01.

Drought stress significantly increased GSH and Pro contents in both flag leaves and roots (*p* < 0.01). Rotation patterns were significantly lower than C1 under both water conditions (*p* < 0.05), with smaller drought-induced increments. For flag leaf GSH, C1 showed the largest drought increment (49.79%); R1 and R2 increased by 17.10% and 15.99%, while R3 showed no significant change between NC and HC, indicating the strongest mitigation (Figure 7A). For root GSH, R2 showed no significant difference between NC and HC, providing the best mitigation (Figure 7B). For flag leaf Pro, C1 increased by 63.47% under drought, while R1 increased only 17.53%, giving the highest drought mitigation effect of all traits at 72.38% (Figure 8A). For root Pro, R2 showed the smallest increment (18.53% vs. 61.06% in C1), with a mitigation effect of 69.65% (Figure 8B). The combined patterns of GSH and Pro indicate that R1 has greater advantage in leaf osmotic protection, while R2 performs better in root osmotic regulation (Table 3).

### 3.6. Two-Way ANOVA

Two-way ANOVA (Table 4) showed that drought (D) and rotation (R) each had highly significant main effects on ROS, MDA, SOD, POD, GSH, and Pro contents in both flag leaves and roots (*p* < 0.01). The D × R interaction was highly significant for flag leaf ROS, MDA, GSH, and Pro (*p* < 0.01) and significant for flag leaf SOD and POD (*p* < 0.05). For root traits, the interaction was highly significant for MDA, GSH, and Pro (*p* < 0.01). Overall, most flag leaf traits and root MDA, GSH, and Pro showed significant D × R interactions, whereas root ROS, SOD, and POD did not, suggesting organ-specific regulation of the antioxidant system by rotation.

### 3.7. Correlations Between Root, Physiological, and Yield-Related Traits

**Figure 9:**
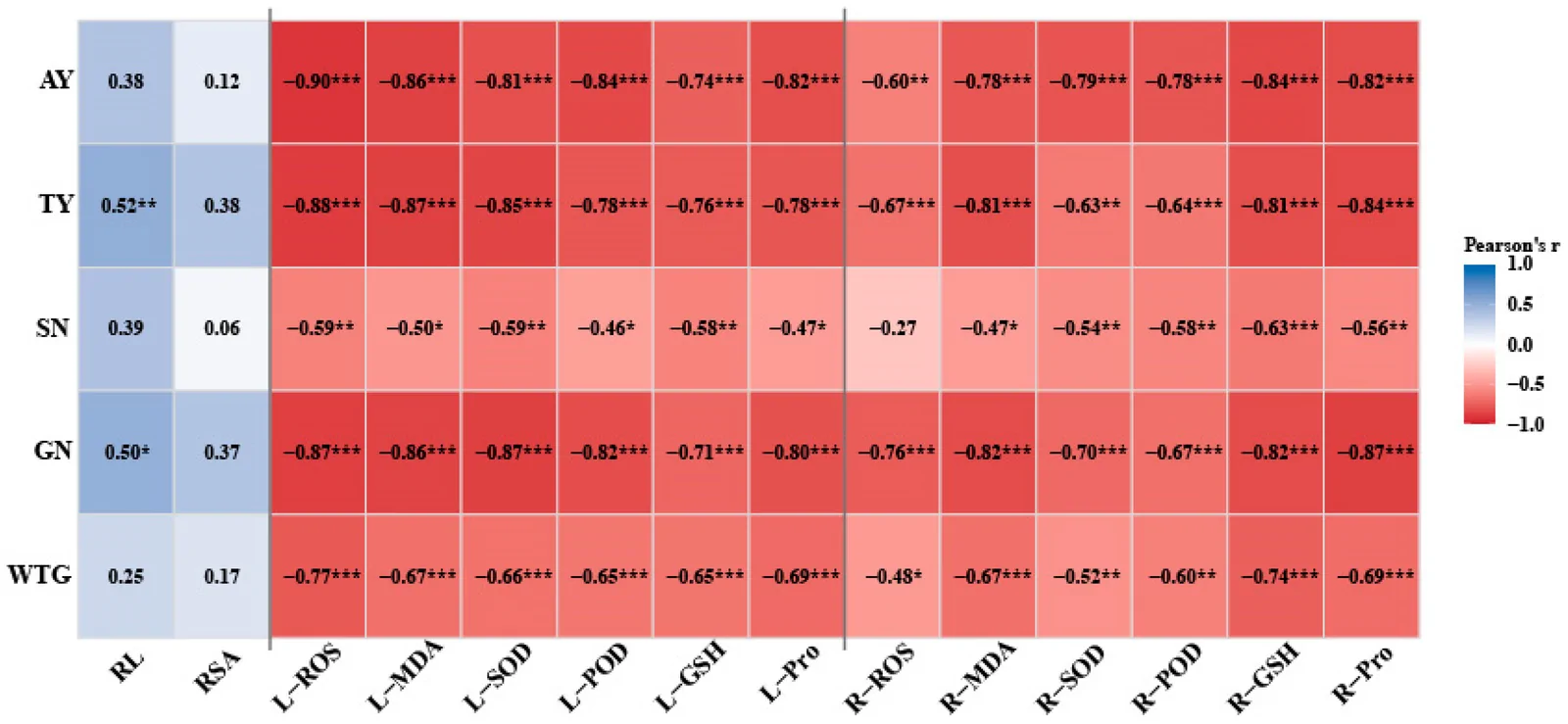
Pearson correlations between root morphological and physiological traits and yield-related traits of spring wheat. Correlations were calculated using 24 plot-level biological observations. Values in the cells represent Pearson’s correlation coefficients (r). Blue and red indicate positive and negative correlations, respectively, with color intensity reflecting the magnitude of the correlation coefficient. *, **, and *** indicate significant correlations at *p* < 0.05, *p* < 0.01, and *p* < 0.001, respectively. RL, root length; RSA, root surface area; L- and R-, flag leaf and root, respectively; ROS, ROS-related levels; MDA, malondialdehyde; SOD, superoxide dismutase; POD, peroxidase; GSH, reduced glutathione; Pro, proline; AY, actual yield; TY, theoretical yield; SN, spike number per unit area; GN, grain number per spike; WTG, 1000-grain weight.

Pearson correlation analysis revealed distinct associations between root and physiological traits and yield-related traits (Figure 9). Root length was positively correlated with theoretical yield (r = 0.52, *p* < 0.01) and grain number per spike (r = 0.50, *p* < 0.05), whereas root surface area was not significantly correlated with any of the five yield-related traits. In contrast, most physiological indicators in both flag leaves and roots were negatively correlated with actual yield, theoretical yield, grain number per spike, and 1000-grain weight. Actual yield showed particularly strong negative correlations with leaf ROS-related levels (r = −0.90, *p* < 0.001), leaf MDA (r = −0.86, *p* < 0.001), leaf POD (r = −0.84, *p* < 0.001), root GSH (r = −0.84, *p* < 0.001), and root Pro (r = −0.82, *p* < 0.001). Grain number per spike was also strongly negatively correlated with leaf ROS-related levels and leaf SOD (both r = −0.87, *p* < 0.001), as well as root Pro (r = −0.87, *p* < 0.001). Compared with the other yield-related traits, spike number generally showed weaker correlations with physiological indicators. Overall, physiological traits exhibited stronger associations with yield-related traits than root morphological traits.

## 4. Discussion

### 4.1. Regulation of Growth and Yield by Rotation Under Drought

Drought curtails crop growth and yield by suppressing cell turgor, photosynthesis, and assimilate accumulation [2,3,5]. In this study, drought significantly reduced spring wheat plant height, dry matter accumulation, root length, and yield, while rotation alleviated these adverse effects. This aligns with the broader understanding that rotation can improve soil health and cropping-system productivity [17], although the magnitude and direction of rotation-associated responses varied among traits and water conditions. Root surface area, however, showed an opposite trend across water conditions: under normal watering, continuous cropping produced a higher root surface area than rotation, whereas under drought, rotation surpassed continuous cropping. A plausible explanation is that the less favorable soil environment under continuous cropping may induce compensatory root proliferation under adequate moisture to sustain water and nutrient acquisition, although such proliferation may not necessarily indicate greater functional efficiency. Under drought, the reduced root surface area under continuous cropping may reflect a lower capacity to maintain this compensatory growth, whereas the more stable root surface area under rotation may be associated with differences in the soil–root environment resulting from long-term rotation history. Yield-component analysis showed that the higher grain number per spike and 1000-grain weight under rotation were important contributors to the yield increase. The 73–78% yield advantage of rotation over continuous cropping partly reflects the depressed yield baseline of C1 under long-term continuous cropping; therefore, this difference captures both the beneficial effects associated with rotation and the negative effects of long-term continuous cropping. The maintenance of growth and yield under rotation may also be associated with coordinated regulation of antioxidant defense in flag leaves and roots. Lower ROS-related levels and lipid peroxidation under rotation indicate reduced oxidative damage, which may contribute to the maintenance of physiological function and assimilate production under drought and thereby support yield stability. Differences in soil water dynamics among rotation histories may themselves constitute one component of the long-term rotation legacy. However, because the present experiment was not designed to separate the direct effects of rotation history from those mediated through soil water dynamics, the observed plant responses should be interpreted as integrated responses associated with long-term rotation history and its accompanying soil water processes. Because this study did not directly measure soil physicochemical properties, microbial community structure, or plant water status, the specific mechanisms underlying the effects of rotation on drought responses require further investigation through integrated soil–plant monitoring.

### 4.2. Regulation of ROS and MDA by Rotation Under Drought

The protective effect of rotation on yield and growth is likely tied to its alleviation of oxidative damage. Czarnocka and Karpinski [26] showed that excess ROS during stress is toxic to plants but also activates defense systems, which attenuate oxidative stress and regulate ROS homeostasis through enzymatic and non-enzymatic pathways. In this study, rotation patterns experienced less drought impact than continuous cropping. This pattern is consistent with greater drought resilience under rotation. MDA, a product of stress-induced membrane lipid peroxidation, is a key indicator of cellular membrane damage—higher MDA signals greater injury [27,28]. Drought significantly increased MDA in flag leaves and roots across all patterns, consistent with reports in *Maclura pomifera* [29] and maize [30]. Rotation markedly reduced MDA content and its drought-induced increment in both organs. This reduction is consistent with lower lipid peroxidation and membrane damage. Under drought, rotation lowered leaf MDA by roughly 30–50% and root MDA by 35–45% relative to continuous cropping. Gong et al. [18] reported that intercropping reduced leaf MDA in proso millet, and Wang et al. [31] observed similar lipid peroxidation mitigation in an eggplant–garlic intercropping system; however, those studies addressed continuous cropping obstacles or intercropping rather than rotation–drought interactions, and involved different crop systems, making direct quantitative comparison difficult. Field evidence on how long-term rotation affects leaf and root MDA responses to drought remains scarce; this study adds new data, though more environments and cultivars are needed for comparison. The mechanism may involve rotation-induced changes in the soil–plant environment that improve plant water and nutrient status and thereby alleviate oxidative stress. Without direct measurements of soil organic matter, nutrients, aggregate structure, or plant water parameters (relative water content, water potential), this mechanism remains speculative. It should also be noted that ROS-related levels in this study were determined using a commercial ELISA kit. Because this approach differs in detection principle from methods such as NBT/DAB staining or fluorescent-probe assays, the resulting values should be interpreted as ROS-related levels rather than direct measurements of instantaneous concentrations of specific ROS. Future studies combining complementary ROS detection methods would allow a more detailed characterization of the effects of rotation on oxidative responses under drought.

### 4.3. Regulation of the Antioxidant Enzyme System by Rotation Under Drought

Lower ROS-related levels and MDA under rotation point to lighter oxidative stress, which should also be reflected in the antioxidant enzyme response. The enzymatic system—SOD, POD, and allied enzymes—scavenges excess ROS [32] and prevents lipid peroxidation, protecting plant cells. In this study, drought significantly increased SOD and POD activities in both leaves and roots across all patterns, consistent with the general finding that drought activates the wheat antioxidant enzyme system [33]. Caverzan et al. [33] reported that drought raises lipid peroxidation and MDA in wheat. POD, a key H_2_O_2_-scavenging enzyme, rises in response to stress as part of the antioxidant defense [32,34]; its increase reflects enhanced ROS-scavenging capacity and adaptive response to drought, helping limit membrane damage and maintain cellular structure and function. Importantly, all rotation patterns were sown to the same cultivar (‘Longmai 36’) in 2022, so differences in antioxidant enzyme activity reflect the soil environment shaped by long-term rotation rather than cultivar differences. Rotation produced smaller drought-induced increments in SOD and POD than continuous cropping. Two non-exclusive explanations are plausible. First, given the concurrent reduction in ROS-related levels and MDA under rotation, the smaller enzyme increment may reflect lighter oxidative stress—plants need not upregulate antioxidant enzymes substantially to maintain redox balance, indicating stronger drought adaptation, possibly associated with differences in the soil–root environment and lower oxidative stress under rotation. Second, long-term continuous cropping may accumulate harmful substances (e.g., phenolic autotoxins or pathogen metabolites) that trigger stress antioxidant responses even under normal watering, artificially elevating the continuous-cropping enzyme baseline; rotation removes this extra oxidative stressor, lowering the baseline. Both mechanisms may contribute jointly. Without measurements of soil toxic substances and microbial community structure, their relative contributions remain to be disentangled.

### 4.4. Regulation of Osmotic Regulators by Rotation Under Drought

Beyond enzymatic antioxidants, non-enzymatic antioxidants and osmotic regulators play a critical role in maintaining cellular homeostasis. Reduced glutathione (GSH) is an important antioxidant that scavenges free radicals and ROS, protecting cells from oxidative damage. During wheat leaf development, GSH helps resist environmental oxidative pressure and maintain cellular homeostasis. GSH concentration shifts with external conditions, particularly under abiotic stress [35,36]. In this study, drought raised GSH in both leaves and roots across all patterns, but rotation showed smaller increments and lower overall levels than continuous cropping, indicating lighter oxidative stress and reduced reliance on antioxidant regulation.

Osmotic regulators participate in drought adaptation by lowering cell osmotic potential, maintaining turgor and water status [37]. In this study, drought raised proline in both leaves and roots across all patterns, consistent with reports in various plants [38] and potato [39] under abiotic stress. Proline increments under rotation were smaller than under continuous cropping; both the absolute proline content and its drought-induced increase were lower under rotation, suggesting weaker osmotic regulation demand under drought. This may be associated with differences in soil water status and plant water availability among rotation histories. Because stomatal regulation plays a central role in coordinating carbon assimilation and water loss, changes in plant water status may also influence water-use efficiency [7]. The higher proline under continuous cropping likely reflects stronger water stress and a correspondingly stronger osmotic regulation response; however, proline accumulation alone is not a reliable indicator of drought tolerance [40].

### 4.5. Organ Specificity of Rotation Regulation

Rotation’s regulation of drought responses differed between leaves and roots. Two-way ANOVA showed that the D × R interaction was significant for most flag leaf traits (ROS-related levels, SOD, POD, etc.) but not for root ROS-related levels, SOD, or POD (Table 4), indicating that rotation’s regulation of the aboveground antioxidant system is more water-dependent, while its regulation of roots is relatively stable and less affected by drought intensity. Several factors may underlie this organ difference. First, leaves directly bear transpirational water loss and photoinhibition, making them more sensitive to atmospheric and soil moisture, whereas roots, buried in soil, respond more to the persistent influence of the rhizosphere environment. Second, root exudates and residue decomposition products from different preceding crops (potato, rape) differentially shape soil microbial communities (e.g., arbuscular mycorrhizal fungi, *Bacillus*), which may sustainably influence wheat root antioxidant baselines via rhizosphere-microbe-mediated induced systemic resistance (ISR)—a reasonable hypothesis pending future verification, as this study did not measure soil microbial communities—while leaves respond more to aboveground signals (e.g., ABA, ROS waves) reflecting instantaneous moisture changes. Third, as the water-absorbing organ, roots may buffer drought through phenotypic plasticity (root length, surface area) before needing to upregulate antioxidant enzymes, reducing the demand for large enzyme increases. This organ difference is also reflected in the mitigation effect values: of the seven entries marked “—” (not applicable) in Table 3 because drought-induced changes were non-significant, five occurred in root traits (root ROS-related levels for R2/R3, root MDA for R1/R2, root GSH for R2) and only two in leaf traits (leaf MDA for R1, leaf GSH for R3), indicating that rotation’s protection of roots is more thorough, reducing drought impact on root traits to non-significant levels under some patterns. In terms of mitigation efficacy, R1 was more effective for leaf traits (ROS-related levels, MDA, Pro), while R2 excelled in root osmotic regulation (root Pro, GSH), suggesting that different rotation patterns show organ-specific regulatory preferences.

Taken together, rotation effectively alleviated the adverse effects of drought by lowering ROS-related levels accumulation and lipid peroxidation and coordinating the response intensity of the antioxidant enzyme system and osmotic regulators, thereby maintaining redox balance and osmotic homeostasis in spring wheat. Recent evidence in spring wheat has shown that drought can alter root exudates and recruit beneficial rhizosphere microorganisms, including *Pseudomonas* and *Streptomyces*, which may contribute to enhanced drought resistance [16]. However, whether long-term rotation modifies these rhizosphere-mediated processes remains to be determined [19]. Among the patterns tested, wheat–potato and wheat–potato–rape rotation showed stronger comprehensive drought resistance.

### 4.6. Associations Between Physiological Responses and Yield Formation

The correlation analysis further linked the physiological responses observed at anthesis with yield formation. Most physiological indicators in both flag leaves and roots were negatively correlated with actual yield, theoretical yield, grain number per spike, and 1000-grain weight, whereas root length was positively associated with theoretical yield and grain number per spike. In particular, actual yield showed strong negative correlations with leaf ROS-related levels and MDA. This pattern is consistent with the general understanding that drought-induced oxidative stress and membrane lipid peroxidation are closely associated with impaired cellular function, restricted crop growth, and reduced productivity [5,26,33,40]. Recent evidence in spring wheat has also shown coordinated drought responses involving oxidative stress, osmotic adjustment, and reductions in plant growth [16].

The negative correlations of SOD, POD, GSH, and Pro with yield should not be interpreted as evidence that these protective enzymes or metabolites adversely affect yield. Rather, their elevated levels likely reflect stronger stress-induced defensive responses. SOD and POD are important components of the enzymatic antioxidant system activated in response to enhanced oxidative pressure [32,33,34], whereas GSH contributes to non-enzymatic redox regulation under water stress [35,36]. Proline accumulation is likewise a well-established component of osmotic adjustment under drought [37,38,40]. Therefore, plots experiencing stronger drought-associated physiological disturbance may simultaneously exhibit greater antioxidant and osmotic responses and lower yield. This interpretation is consistent with the present treatment responses, in which continuous cropping generally showed stronger physiological responses and lower yield, whereas rotation treatments showed smaller drought-induced physiological changes and better yield maintenance.

Grain number per spike showed particularly strong correlations with both leaf and root physiological indicators, whereas spike number was generally less strongly correlated. This pattern may reflect a closer association between physiological status around anthesis and subsequent grain formation and filling than with the earlier establishment of spike number. Root length was positively correlated with theoretical yield and grain number per spike, suggesting that maintenance of root growth under water limitation was associated with improved yield formation. By contrast, root surface area was not significantly correlated with yield-related traits, indicating that individual root morphological characteristics do not necessarily translate directly into final yield performance.

It should be emphasized that the correlation analysis combined observations across both water regimes and all rotation patterns. Therefore, the strong correlations partly reflect common variation associated with drought treatment and long-term rotation history and should be interpreted as associations rather than direct causal relationships. Nevertheless, these results provide complementary evidence that lower oxidative stress-related levels and more moderate antioxidant and osmotic responses under rotation were closely associated with improved yield performance, particularly grain number and grain weight.

### 4.7. Limitations and Prospects

This study has several limitations that should be addressed in future work. First, physiological and agronomic measurements were conducted primarily in 2022, and physiological indicators were measured at a single growth stage (anthesis), resulting in a lack of multi-year and multi-stage dynamic data (e.g., jointing, grain filling, and milk ripening) to evaluate the temporal stability of rotation effects. In addition, the lack of complete site-specific meteorological records for the 2016–2021 rotation-establishment period limited our ability to evaluate the potential influence of interannual climatic variability on long-term rotation effects. Second, soil physicochemical properties (organic matter, nutrients, enzyme activities), microbial community structure, and crop water-use efficiency were not directly measured; the mechanistic inference that rotation mitigates drought by improving the rhizosphere rests largely on logical deduction and lacks direct evidence. Third, the rotation cycles differ in length (R1/R3 = 2 years; R2 = 3 years), so by 2022 the patterns had experienced different numbers of wheat seasons (R1/R3 = 4; R2 = 3), potentially confounding rotation frequency with rotation composition. Fourth, the study used a single cultivar (Longmai 36) at a single site; extrapolation requires multi-cultivar, multi-site validation. Fifth, the yield differences among rotation patterns reflect both the positive effect of rotation and the negative effect of continuous cropping obstacles; the current design cannot fully separate the two, and future work could add rotation vs. fallow or rotation vs. first-crop controls to disentangle them.

## 5. Conclusions

Long-term rotation alleviated the adverse effects of drought on spring wheat growth, physiological responses, and yield. Under drought, actual yields of R1 and R2 were 73.26% and 77.67% higher than that of continuous cropping, respectively, although these relative increases partly reflected the depressed yield baseline under long-term continuous cropping. Rotation was also associated with lower ROS-related levels and MDA levels and smaller drought-induced changes in antioxidant enzymes, GSH, and Pro, indicating reduced oxidative damage and a lower requirement for stress-induced antioxidant and osmotic adjustment. R1 showed stronger mitigation of several leaf responses, whereas R2 showed greater advantages in root osmotic-regulation indicators.

Correlation analysis further showed that most leaf and root physiological indicators were negatively associated with actual yield, theoretical yield, grain number per spike, and 1000-grain weight, whereas root length was positively associated with theoretical yield and grain number per spike. These relationships indicate that lower physiological stress intensity was closely associated with improved yield performance, particularly with grain formation and grain weight. Because these correlations incorporate variation caused by both water regime and rotation history, they should be interpreted as associations rather than causal relationships. Overall, the results demonstrate that long-term rotation, particularly wheat–potato and wheat–potato–rapeseed rotations, has strong potential to improve spring wheat drought resilience through integrated effects on growth, physiological responses, and yield formation. Multi-year and multi-site studies integrating soil, plant–water, and rhizosphere measurements are needed to further resolve the underlying mechanisms.

## Figures and Tables

**Table 1:** Two-way ANOVA of agronomic traits and root phenotype in spring wheat (F values).

Source	df	Plant Height	Fresh Weight	Dry Weight	Root Length	Root Surface Area
Drought (D)	1	58.23 **	116.50 **	75.87 **	72.88 **	24.58 **
Rotation (R)	3	45.99 **	11.91 **	23.91 **	0.59	0.18
D × R	3	0.40	0.24	0.04	8.52 **	7.51 **

Note: ** indicates a highly significant effect (*p* < 0.01). Each treatment combination consisted of three independent biological replicates (n = 3).

**Table 2:** Two-way ANOVA of yield and yield components in spring wheat (F values).

Source	df	Actual Yield	Theoretical Yield	Spike Number	Grain Number	1000-Grain Weight
Drought (D)	1	31.80 **	53.20 **	3.39	86.43 **	1.52
Rotation (R)	3	42.08 **	37.18 **	5.56 **	39.44 **	9.91 **
D × R	3	0.29	0.45	0.56	0.14	0.31

Note: ** indicates a highly significant effect (*p* < 0.01). Each treatment combination consisted of three independent biological replicates (n = 3).

**Table 3:** Summary of drought mitigation effect values for each physiological trait under different rotation patterns (%).

Indicator	Organ	R1 (%)	R2 (%)	R3 (%)
ROS	Leaf	55.23	34.95	42.72
ROS	Root	8.95	—	—
MDA	Leaf	—	25.59	18.76
MDA	Root	—	—	56.93
SOD	Leaf	44.86	58.71	60.90
SOD	Root	41.17	36.38	19.36
POD	Leaf	28.42	48.88	34.34
POD	Root	48.30	23.24	30.27
GSH	Leaf	65.65	67.89	—
GSH	Root	21.89	—	59.15
Pro	Leaf	72.38	65.81	47.24
Pro	Root	57.20	69.65	38.83

Note: Drought mitigation effect values were calculated from treatment means based on three independent biological replicates (n = 3) per treatment. “—” indicates that the drought mitigation effect value was not applicable because the difference between NC and HC was not significant (*p* > 0.05).

**Table 4:** Two-way ANOVA of physiological traits in flag leaves and roots of spring wheat (F values).

Position	Source of Variation	df	ROS	MDA	SOD	POD	GSH	Pro
Leaf	Drought stress (D)	1	169.23 **	117.92 **	259.06 **	103.92 **	93.86 **	262.77 **
Leaf	Rotation pattern (R)	3	316.12 **	156.33 **	119.48 **	35.43 **	43.14 **	477.05 **
Leaf	D × R	3	21.17 **	12.02 **	25.13 *	3.57 *	16.01 **	58.71 **
Root	Drought stress (D)	1	35.17 **	47.23 **	62.26 **	38.87 **	99.69 **	220.15 **
Root	Rotation pattern (R)	3	9.55 **	79.21 **	15.10 **	23.60 **	134.05 **	168.12 **
Root	D × R	3	2.03	9.01 **	1.39	1.24	16.74 **	29.42 **

Note: * and ** indicate significant (*p* < 0.05) and highly significant (*p* < 0.01) effects, respectively. Each treatment combination consisted of three independent biological replicates (n = 3).

## Data Availability

The original contributions presented in this study are included in the article/Supplementary Material. Further inquiries can be directed to the corresponding author.

## Supplementary Materials

The following supporting information can be downloaded at: https://www.mdpi.com/article/10.3390/plants15162535/s1.
