# Supplementary material for: Long-Term Crop Rotation Improves Drought Resilience and Modulates Antioxidant Responses in Spring Wheat Leaves and Roots
Source: Plants (Basel). 2026 Aug 21;15(16):2535. doi: 10.3390/plants15162535 (PMC13516447; doi:10.3390/plants15162535)
Supplement: Supplementary file 1 [file plants-15-02535-s001.zip › plants-4485081-supplementary.pdf]

Supplementary Table S1. Crop sequence of different rotation patterns from 2016 to 2022

Treatment	2016	2017	2018	2019	2020	2021	2022
R1	Wheat	Potato	Wheat	Potato	Wheat	Potato	Wheat
R2	Wheat	Potato	Rape	Wheat	Potato	Rape	Wheat
R3	Wheat	Rape	Wheat	Rape	Wheat	Rape	Wheat
C1	Wheat	Wheat	Wheat	Wheat	Wheat	Wheat	Wheat

Supplementary Table S2. Crop-specific field management practices during the rotation experiment

Crop	Variety name	Row spacing(cm)	Seeding rate(kg /ha)	Seed spacing(cm)	Fertilizer rate(kg/ha)			
					Nitrogen (CH_4N_2O)	Phosphorus ($(NH_4)_2HPO_4$)	Potassium (K_2SO_4)	Boron ($Na_2B_4O_7 \cdot 10H_2O$)
Wheat	Longmai 36	15	20	/	60	60	30	
Rape	Qingza 5	30	6	/	60	165	30	3
Potato	Xingjia 2	65	/	30	225	225	90	

Supplementary Table S3. Plot-level physiological data of spring wheat flag leaves and roots under different water and rotation treatments

**Treatment**	**Replicate**	**Organ**	**ROS-related levels**	**MDA**	**SOD**	**POD**	**GSH**	**Pro**
NCC1	1	Flag leaf	4.39	36.83	221.44	61.07	2.87	159.32
NCC1	2	Flag leaf	4.29	38.81	204.56	62.09	2.71	143.31
NCC1	3	Flag leaf	5.09	44.58	237.38	59.17	3.03	180.22
NCC1	1	Root	1.86	20.04	163.62	58.18	2.35	16.88
NCC1	2	Root	1.98	21.40	181.78	50.68	2.52	19.00
NCC1	3	Root	1.99	19.99	170.12	69.04	2.69	16.87
NCR1	1	Flag leaf	2.96	34.88	189.10	50.88	2.63	78.42
NCR1	2	Flag leaf	2.33	32.39	177.82	52.56	2.46	85.55
NCR1	3	Flag leaf	2.94	31.14	170.42	51.87	2.58	100.33
NCR1	1	Root	1.64	10.56	144.44	45.64	1.90	10.11
NCR1	2	Root	1.79	14.84	167.24	58.57	1.81	13.24
NCR1	3	Root	1.87	15.79	173.48	54.97	2.14	10.63
NCR2	1	Flag leaf	2.80	24.90	157.43	47.27	2.41	64.49
NCR2	2	Flag leaf	2.39	29.90	186.01	53.15	2.85	85.76
NCR2	3	Flag leaf	2.12	25.35	175.66	53.99	2.82	73.87
NCR2	1	Root	1.75	13.77	135.18	40.20	1.77	13.10
NCR2	2	Root	1.68	13.26	157.49	51.73	1.83	13.77
NCR2	3	Root	2.05	18.01	158.87	51.91	2.16	14.00
NCR3	1	Flag leaf	2.39	22.68	189.93	58.92	2.86	108.17
NCR3	2	Flag leaf	2.72	29.33	175.60	50.37	2.52	123.53

NCR3	3	Flag leaf	2.93	21.08	169.35	50.83	1.96	115.74
NCR3	1	Root	1.72	12.91	169.33	53.10	1.61	13.36
NCR3	2	Root	1.86	18.24	124.98	41.06	1.35	11.04
NCR3	3	Root	1.97	15.94	152.29	48.34	1.88	12.19
HCC1	1	Flag leaf	6.43	54.19	337.44	80.90	4.43	273.96
HCC1	2	Flag leaf	6.63	51.55	327.61	76.40	4.48	261.13
HCC1	3	Flag leaf	6.73	55.87	345.77	74.56	3.99	254.21
HCC1	1	Root	2.17	25.60	188.83	68.65	3.52	26.50
HCC1	2	Root	2.15	29.06	238.14	77.54	3.55	27.91
HCC1	3	Root	2.36	30.26	209.99	70.98	3.40	30.55
HCR1	1	Flag leaf	3.31	35.47	205.78	61.74	2.61	96.01
HCR1	2	Flag leaf	3.34	32.84	232.25	68.38	3.11	110.73
HCR1	3	Flag leaf	3.19	36.97	254.48	55.40	3.27	103.90
HCR1	1	Root	2.10	16.77	172.78	51.63	2.36	16.73
HCR1	2	Root	1.90	12.35	190.58	64.77	2.87	13.91
HCR1	3	Root	2.00	17.13	189.03	60.93	2.37	12.22
HCR2	1	Flag leaf	2.90	32.78	204.68	57.32	2.89	102.44
HCR2	2	Flag leaf	3.24	38.36	192.25	51.92	3.56	76.00
HCR2	3	Flag leaf	3.25	29.54	234.48	66.62	2.93	94.34
HCR2	1	Root	2.08	16.46	157.50	49.90	1.94	16.15
HCR2	2	Root	1.86	16.40	179.84	60.44	1.87	16.76
HCR2	3	Root	1.93	16.81	181.89	57.85	2.22	15.54
HCR3	1	Flag leaf	3.57	27.50	209.03	62.79	3.17	163.80
HCR3	2	Flag leaf	3.24	33.94	196.60	69.44	2.51	154.18
HCR3	3	Flag leaf	3.25	32.09	238.83	56.45	2.48	145.80
HCR3	1	Root	2.03	18.33	186.58	61.09	1.71	18.42
HCR3	2	Root	1.97	20.16	190.85	54.74	1.94	14.50
HCR3	3	Root	1.83	16.36	154.03	48.60	1.95	17.34

Note: Each treatment consisted of three independent biological replicates (n = 3). Flag leaf and root samples from 15 plants within each plot were pooled as one biological replicate.
